# The effect of CBT-based journaling on emotional responses to clothing styles

**DOI:** 10.1097/MD.0000000000050018

**Published:** 2026-08-07

**Authors:** Ekwutosi Beatrice Oluah, Uzoamaka C. Ogor, Damian C. Ncheke, Lilian C. Ozoemena, Ngozi Ngwanma Awoke, Uche Hope Ekwueme, Nkechi T. Egenti, Dorathy C. Ezeunara, Chinonye Lilian Opara, Patience Nwobodo, Blessed Samuel Okechukwu Efido, Daniel Okon

**Affiliations:** a Department of Home Science & Management, University of Nigeria, Nsukka, Nigeria; b Department of Mass Communication, University of Nigeria, Nsukka, Nigeria; c Department of Counseling and Human Development Studies, University of Nigeria, Nsukka, Nigeria; d Department of Guidance and counselling Alex Ekwueme Federal University, Abakaliki, Ebonyi State, Nigeria; e Department of Educational Foundations, University of Nigeria, Nsukka, Nigeria; f Department of Education, Federal College of Education (Technical), Isu, Ebonyi State, Nigeria; g Department of Art Education, University of Nigeria, Nsukka, Nigeria; h Department of Clinical Counselling, University of Bamenda, Bamenda, Northwest Region, Cameroon.

**Keywords:** adolescents, CBT-based journaling, clothing styles, emotional response, irrational beliefs

## Abstract

**Background:**

Clothing styles influence both outward appearance and internal emotional responses. Adolescents and young adults are particularly susceptible to emotional responses and irrational beliefs related to clothing styles. However, there is limited evidence on the efficacy of cognitive-behavioral therapy (CBT)-based journaling interventions in addressing these issues among non-clinical populations. This study examined the effects of CBT-based journaling on emotional responses and irrational beliefs related to clothing styles.

**Methods:**

A randomized controlled trial involving 128 adolescents and young adults aged 15 to 22 years was conducted in Nsukka, Nigeria. Participants were randomly assigned to either a CBT-based journaling intervention group (n = 63) or a control group (n = 65). The 6-week intervention consisted of structured journaling targeting cognitive restructuring, emotional responses, and irrational beliefs related to clothing styles. Baseline and post-intervention assessments utilized the Difficulties in Emotion Regulation Scale for Clothing Styles and the Clothing Style Automatic Thoughts Questionnaire. Data were analyzed using ANCOVA, controlling for baseline scores.

**Results:**

The intervention group demonstrated significant improvement in emotional regulation compared with the control group, *F*(1, 125) = 253.10, *P* < .001, partial η² = 0.669. Similarly, the intervention group exhibited significantly reduced irrational beliefs, *F*(1, 125) = 127.82, *P* < .001, partial η² = 0.506. In both cases, CBT-based journaling accounted for a large proportion of the variance in post-intervention outcomes.

**Conclusion:**

CBT-based journaling is an effective and scalable intervention for improving emotional responses and reducing irrational beliefs related to clothing styles. Its efficacy highlights the potential for broad application in mental health promotion, particularly in appearance-sensitive contexts among adolescents and young adults.

## 1. Introduction

Clothing styles influence individual identity, self-perception, and emotional expression. Beyond their utilitarian purpose, garments act as a medium through which individuals convey their personality, values, mood, and social connections.^[1]^ Nevertheless, the emotional responses to clothing choices are not invariably positive. For numerous individuals, the act of selecting or donning specific styles can trigger feelings of anxiety, self-consciousness, or distress, particularly when shaped by internalized societal norms or skewed self-assessments.

Emotional responses to clothing styles pertain to the affective experiences that individuals encounter when choosing, wearing, or being observed in certain clothing types.^[2–5]^ These emotions can vary from confidence, joy, and pride to shame, anxiety, and self-doubt, contingent upon the wearer’s internal associations with specific clothing styles and their perceptions of others’ judgments.^[3,6]^ Worldwide, the impact of clothing on emotional well-being is increasingly recognized, especially as individuals navigate sociocultural standards of appearance in diverse environments such as educational institutions, workplaces, and social media platforms.^[7,8]^ For example, research revealed that clothing styles are intricately linked to body satisfaction and mood in both adolescents and adults, with many experiencing emotional distress when their clothing does not align with internal or external expectations.^[7,8]^ In societies where appearance is closely associated with social status or gender roles, clothing-related emotional distress may be more pronounced.^[9–11]^ Consequently, emotional responses to clothing extend beyond mere stylistic preferences; they serve as indicators of more profound psychological and social dynamics, influencing confidence, identity, and mental health.

Closely associated with these emotional responses are irrational beliefs concerning clothing styles, rigid, exaggerated, or illogical notions that affect how individuals assess themselves and others based on attire. These beliefs may encompass absolutist thoughts such as “I must always dress impeccably to gain respect” or “People will only appreciate me if I don designer labels.” Typically unconscious, these beliefs are bolstered by societal expectations, peer influences, and media representations of idealized appearances.^[12–16]^ Research shows that irrational beliefs regarding clothing and appearance are widespread across various demographics, particularly among adolescents and young adults who are more prone to cognitive distortions related to identity.^[10,17]^ Such beliefs can lead to persistent dissatisfaction, anxiety related to appearance, and maladaptive behaviors like compulsive shopping or social withdrawal. If not addressed, they may contribute to more extensive psychological challenges, including body image issues, diminished self-worth, and symptoms of depression.^[18]^ Thus, recognizing and altering these irrational beliefs is essential for enhancing individuals’ emotional responses and fostering healthier relationships with clothing styles.

The present study focuses on adolescents and young adults largely drawn from Generations Z and Alpha, cohorts whose developmental experiences are strongly shaped by digital media, visual self-presentation, and continuous social evaluation.^[19,20]^ Generation Z generally includes individuals born between about 1997 and 2012, who grew up alongside smartphones and social networking platforms where appearance, identity performance, and constant comparison have become part of everyday life.^[19,20]^ Generation Alpha, born from around 2013 onward, is the first generation to experience digital technology from early childhood, with exposure to influencer culture, visual consumption norms, and rapid online feedback shaping emotional development and self-concept formation.^[21]^

Research increasingly shows that these generations display greater emotional sensitivity to self-presentation, stronger connections between appearance and self-worth, and a higher tendency toward irrational beliefs related to social approval and perceived judgment.^[19,20]^ The constant stream of visual feedback on social media intensifies how young people interpret clothing choices, body image, and social status, often leading to heightened emotional reactions and distorted self-evaluations.^[21,22]^ For Generation Alpha in particular, early immersion in carefully curated digital environments appears to accelerate self-comparison processes and increase vulnerability to emotional dysregulation and anxiety-related concerns.^[21]^

Within this broader developmental and social context, emotional responses to clothing styles are more than simple aesthetic preferences. They function as psychologically meaningful experiences that influence identity formation, self-esteem, and emotion regulation.^[19]^ Irrational beliefs, such as equating social acceptance with perfect appearance or viewing clothing choices as measures of personal worth, are especially common among digitally immersed youth and often reinforce cycles of emotional distress.^[20]^ As a result, interventions that directly address cognitive restructuring and emotional regulation, including CBT-based journaling, are particularly suitable for Generation Z and Alpha populations navigating appearance-focused social environments.

Among students especially adolescents and young adults, the convergence of emotional responses to clothing styles and irrational beliefs about appearance is particularly significant. This demographic is at a developmental phase where identity formation, peer acceptance, and social comparison are highly pronounced, rendering them especially susceptible to pressures related to appearance. Students frequently encounter both internal and external demands to conform to prevailing fashion trends, body ideals, or the norms of their peer groups. When they perceive themselves as inadequate, they may experience intense emotional responses such as embarrassment, anxiety, or diminished self-worth.^[18]^ Moreover, the emergence of image-focused platforms such as Instagram, TikTok, and Snapchat has exacerbated these pressures, cultivating a culture in which clothing and appearance are closely tied to social acceptance.^[23]^ Research indicates that a considerable proportion of students report feeling distress when they perceive an inability to afford, fit into, or adequately express themselves through trendy clothing styles.^[24]^ This distress is often fueled by deeply ingrained irrational beliefs, such as the necessity to conform to a specific appearance for acceptance or the belief that others are perpetually evaluating their looks, which can hinder academic involvement, heighten social withdrawal, and lead to adverse mental health effects like anxiety and depression.^[25]^ In light of these trends, there is an urgent demand for accessible, evidence-based interventions that assist students in recognizing and challenging these irrational beliefs, as well as managing their emotional responses to experiences related to clothing styles.

Cognitive Behavioral Therapy (CBT)-based journaling presents a practical, organized, and psychologically sound approach for tackling the maladaptive emotional and cognitive responses that students frequently encounter concerning clothing styles.^[26–28]^ Grounded in CBT principles, this journaling method entails guided self-reflection that aids individuals in identifying and disputing irrational beliefs, exploring the emotional repercussions of those beliefs, and substituting them with more balanced, reality-oriented thoughts.^[29]^ In contrast to general expressive writing, CBT-based journaling is specifically crafted to reformulate distorted thinking patterns through strategies such as thought records, cognitive reframing, and behavioral reflection. For students navigating intricate social settings where their appearance is subject to intense scrutiny, this approach offers a confidential, non-judgmental environment to reflect on their experiences, assess the validity of their beliefs, and cultivate healthier emotional responses to clothing-related stimuli. Studies have indicated that CBT journaling can alleviate anxiety symptoms, boost self-esteem, and improve emotional regulation by allowing individuals to articulate and critically evaluate their automatic thoughts.^[30,31]^ Furthermore, it is a cost-effective method that can be seamlessly incorporated into school wellness initiatives, empowering students to actively manage their mental health.^[32,33]^ Despite its potential benefits, there is a notable scarcity of empirical research applying CBT-based journaling specifically to issues surrounding clothing, fashion identity, or self-assessments based on appearance.^[34]^ Therefore, exploring its impact in this domain could address a significant gap in the existing literature while providing a scalable solution to a prevalent psychological issue among students.^[35]^

Given the psychological susceptibility of students to pressures related to appearance and the increasing influence of clothing on emotional health, there is an urgent demand for empirically validated interventions that address both emotional responses and the irrational beliefs that underpin them.^[1,28]^ CBT-based journaling is particularly noteworthy as a promising resource due to its accessibility, flexibility, and evidence-based grounding in cognitive restructuring.^[36,37]^ Nevertheless, its application to emotional challenges associated with clothing remains insufficiently examined in empirical studies.^[38,39]^ This research, therefore, aims to fill this void by exploring the effects of CBT-based journaling on students’ emotional response to clothing styles and the irrational beliefs related to clothing styles. In doing so, the study aspires to provide substantial evidence for a low-cost, easily implementable intervention that can be integrated into mental health support programs for students. Ultimately, the goal is to foster healthier self-perception, enhance emotional resilience, and promote psychological well-being in a fashion-driven, socially evaluative world.

### Hypotheses

The following null hypotheses were tested:

H_1_: CBT-based journaling has no significant effect on emotional responses to clothing styles.
H_2_: CBT-based journaling has no significant effect on irrational beliefs related to clothing styles.

## 2. Methods

### 2.1. Design

This study adopted a pretest-posttest quasi-experimental design involving an experimental group exposed to a CBT-based journaling intervention and a control group that did not receive any intervention. The design allowed for the measurement of changes in participants’ emotional responses to clothing color and style, as well as automatic thoughts related to clothing, before and after the intervention. This design was chosen because it is effective in evaluating the impact of psychological interventions in naturalistic settings without requiring random assignment.

### 2.2. Participants

A total of 200 students were assessed for eligibility but 70 was excluded (50 did not meet the criteria while 20 declined to participate in the study). The final sample comprised 128 students aged 15 to 22 years, who report unhelpful or distressing thoughts and feelings associated with their clothing styles. Participants were recruited from secondary schools and community centers within the Nsukka Education Zone of Enugu State, Nigeria. Inclusion criteria included: individuals aged 15 to 22 years; self-reported emotional difficulties associated with clothing styles, as indicated by scores within designated ranges, difficulties in emotion regulation scale for clothing styles (DERSCS) (144–180), and CSATQ (120–150); and a voluntary willingness to participate in the study. Exclusion criteria encompassed a confirmed diagnosis of severe mental health conditions or neurological disorders, as verified by school counselors or health documentation, and refusal to provide informed consent or withdrawal from the study after enrollment. Eligible participants were randomly assigned to either the experimental group (which received a CBT-based journaling intervention) or the control group (which received no intervention) using a computerized randomization tool to ensure allocation concealment. Both parental consent and participant assent were obtained prior to participation, in accordance with established ethical standards and institutional review board protocols. A priori power analysis was conducted using G*Power software^[40]^ to determine the minimum required sample size. For a multivariate analysis of variance assessing global effects with 2 groups and 3 dependent variables, utilizing a moderate effect size (*f*^2^(*V*) = 0.0625), α = .05, and power (1 − β) = 0.80, the necessary total sample size was calculated to be 108 participants. To account for potential attrition (estimated at 20%), the target sample size was adjusted to 130 participants (65 per group), thereby ensuring sufficient statistical power to detect main and interaction effects. Efforts were made to achieve gender balance across groups to improve the generalizability of the findings (Insert flow diagram).

## 3. Instruments

### 3.1. The difficulties in emotion regulation scale for clothing styles (DERSCS)

The DERSCS is a context-specific adaptation of the original difficulties in emotion regulation scale (DERS) developed by Gratz and Roemer.^[41]^ The DERSCS was designed to assess how individuals regulate their emotions in relation to clothing styles. While it retains the original 6 subscales – nonacceptance of emotional responses, difficulties engaging in goal-directed behavior, impulse control difficulties, lack of emotional awareness, limited access to regulation strategies, and lack of emotional clarity – the items have been reworded to reflect challenges that arise from clothing-related emotional experiences.

The instrument consists of 36 items rated on a 5-point Likert scale ranging from 1 (“almost never”) to 5 (“almost always”). Higher total scores indicate greater difficulty regulating emotions in relation to clothing and appearance. Sample adapted items include statements such as, *“When I feel uncomfortable in my outfit, I have difficulty controlling my reactions,”* and *“I get upset with myself for feeling bad about how I look in certain clothes.”* Other examples include, *“My emotions about clothing styles interfere with my ability to focus on other tasks,”* and *“I have trouble calming down when I’m upset about my clothing choices.”* These adaptations maintain the structure and diagnostic utility of the original DERS while offering greater sensitivity to appearance-related emotional triggers.

Preliminary psychometric evaluation of the DERSCS indicated excellent internal consistency (Cronbach alpha > .90) and strong face and construct validity. The scale has demonstrated relevance for both clinical and non-clinical settings, particularly in assessing interventions aimed at addressing appearance-related emotional dysregulation. Its sensitivity to treatment-related changes makes it particularly suitable for evaluating the outcomes of cognitive-behavioral therapy (CBT)-based interventions, such as journaling programs focused on reshaping beliefs and emotional responses linked to clothing and fashion choices.

### 3.2. Clothing style automatic thoughts questionnaire (CSATQ)

The clothing style automatic thoughts questionnaire (CSATQ) was developed by adapting the original automatic thoughts questionnaire (ATQ) created by Hollon and Kendall in 1980.^[42]^ The ATQ is a 30-item self-report instrument designed to measure the frequency of negative automatic thoughts typically associated with depression. Respondents rate each item on a 5-point Likert scale ranging from 1 (“Not at all”) to 5 (“All the time”), based on how often they experienced the thought over the past week. Scores range from 30 to 150, with higher scores reflecting a greater frequency of negative self-referential cognitions. The original ATQ has been widely validated and demonstrates excellent psychometric properties, including high internal consistency, with Cronbach alpha values typically around 0.96. Factor analyses have identified key domains such as personal maladjustment, negative self-concept, and pessimistic expectations. The instrument has also shown strong convergent validity with established depression measures and has been effective in differentiating between depressed and non-depressed populations. In the present study, the ATQ was adapted to reflect the specific cognitive and emotional responses associated with clothing and fashion. This newly adapted measure, the CSATQ, retains the original structure and response scale of the ATQ but modifies the item content to focus on negative automatic thoughts related to clothing styles, self-presentation, and perceived social evaluation based on attire. This adaptation allows for a more targeted assessment of fashion-related cognitive distortions, which are particularly relevant in the context of self-expression and emotional regulation through clothing.

Sample items in the CSATQ include statements such as: *“I look ridiculous in this outfit,” “People will judge me negatively because of what I’m wearing,” “No matter what I wear, I never look right,”* and *“I can’t express who I am through my clothing.”* These items reflect the kinds of irrational and self-critical thoughts that may arise in adolescents struggling with self-image, style conformity, or social anxiety related to appearance. The CSATQ is designed to be sensitive to cognitive and emotional shifts following interventions, making it well-suited for evaluating outcomes in this CBT-based journaling study. Preliminary use of the adapted questionnaire in a pilot sample has shown promising results, with strong internal consistency and face validity. As such, the CSATQ provides a reliable and contextually relevant tool for measuring the impact of cognitive-behavioral interventions on clothing-related emotional responses and self-perception.

### 3.3. Procedure

Following approval by the relevant institutional ethics committee, participants were recruited from secondary schools and community centers in the Nsukka Education Zone, Enugu State, Nigeria. Eligibility screening was conducted based on the inclusion and exclusion criteria, and informed consent was obtained from all participants or their legal guardians where appropriate. Participants first completed the baseline measures, including the DERSCS and CSATQ, administered in supervised group sessions.

Participants were then randomly assigned to either the experimental group or the control group using a computerized randomization programme to ensure allocation concealment. The experimental group received a CBT-based journaling intervention, while the control group did not receive any intervention during the study period. The CBT-based journaling intervention spanned 6 weeks and included structured daily entries targeting the identification and modification of negative emotional responses and irrational beliefs about clothing styles. Participants were guided to recognize negative automatic thoughts, apply cognitive restructuring techniques, and document their emotional experiences related to clothing styles.

Research assistants maintained weekly check-ins with participants in the experimental group to monitor adherence and provide support. After 6 weeks, both groups completed the post-intervention assessments using the same instruments as at baseline. Data collection was conducted by research assistants blinded to group assignment to minimize assessment bias. Participant confidentiality was maintained throughout the study, and withdrawal at any point without consequence was emphasized.

### 3.4. Ethical considerations

The study protocol was reviewed and approved by the Research Ethics Committee of the Department of Counselling and Human Development Studies, University of Nigeria, Nsukka, with approval number – REC/UNN/CHDS/2025/000061. All procedures were conducted in accordance with the ethical standards of the Declaration of Helsinki. Written informed consent was obtained from all participants and, where applicable, from their legal guardians. Participants were informed of their right to withdraw from the study at any time without penalty. Confidentiality and anonymity were assured by assigning unique codes to each participant, and all data were securely stored and used solely for research purposes. Furthermore, the study was registered with the Pan African Clinical Trial Registry. The unique identification number for the registry is PACTR202605716561516.

### 3.5. Intervention procedure

The intervention employed in this study was adapted from *A Provider’s Guide to Brief Cognitive Behavioral Therapy* developed by Cully, Dawson, Hamer, and Tharp (2020) under the U.S. Department of Veterans Affairs South Central Mental Illness Research, Education, and Clinical Center.^[43]^ This manual provides a structured, evidence-based approach for brief cognitive behavioral therapy (CBT) interventions. For the present study, it was modified to focus on CBT-based journaling exercises targeting emotional responses to clothing styles.

Participants in the experimental group engaged in a six-week CBT-based journaling program designed to enhance positive affect, reduce negative affect, and challenge irrational beliefs. In the first week, participants were introduced to basic CBT principles and instructed to journal about their daily emotional experiences related to clothing choices, documenting their automatic thoughts without attempting to modify them. In the second week, the journaling tasks focused on behavioral activation, encouraging participants to record positive activities associated with wearing different clothing styles and the resulting emotional responses. Weeks 3 and 4 emphasized cognitive restructuring. Participants identified and challenged irrational beliefs and cognitive distortions surrounding clothing styles, utilizing structured thought records to replace maladaptive thoughts with rational alternatives. In the fifth week, journaling centered on problem-solving, with participants describing obstacles related to clothing choices and documenting adaptive strategies used to address these challenges. The final week of the intervention focused on emotional response and consolidation, where participants reflected on their overall progress, highlighting instances in which they successfully managed negative emotions through cognitive and behavioral techniques.

All journaling prompts were standardized to ensure consistency across participants. Participants submitted their entries weekly, either through secure online platforms or in sealed envelopes, depending on logistical arrangements. The control group did not participate in any structured journaling or CBT-related activities during the study period but received no treatment following the completion of the study. Assessments of emotional response and irrational beliefs were administered at baseline and post-intervention. The intervention procedures were closely monitored to maintain fidelity to the principles outlined in the original CBT manual while ensuring the program remained accessible and developmentally appropriate for adolescents and young adults.

### 3.6. Data analysis

Data were analyzed using International Business Machines SPSS Statistics version 27 (IBM Corp., Armonk). Descriptive statistics, including means and standard deviations, were computed to summarize participants’ scores on the dependent variables across experimental and control groups. Prior to inferential analysis, the data were screened for missing values and outliers, and relevant statistical assumptions for analysis of covariance (ANCOVA) were assessed. These included normality of residuals, linearity, homogeneity of regression slopes, homogeneity of variances (Levene’s test), and the absence of multicollinearity. All assumptions were adequately met.

Subsequently, a series of one-way ANCOVAs were conducted to determine the effect of group (experimental vs control) on each dependent variable (emotional responses and irrational beliefs) while controlling for the corresponding baseline (pre-intervention) scores. This approach allowed for the adjustment of any initial differences between groups prior to the intervention. Partial eta squared (η^2^) was used to estimate effect sizes, and statistical significance was determined at *P* < .05.

## 4. Results

Descriptive statistics in Table 1 were computed to examine the pretest and posttest scores of participants on the DERS and the CSATQ across the treatment and control groups. For participants who received CBT-based journaling (treatment group), the mean DERS score decreased from 152.03 (SD = 7.12) at pretest to 72.44 (SD = 9.25) at posttest, reflecting a mean score reduction of 79.59 points. This score reduction represents a mean gain in emotional regulation. Similarly, their CSATQ scores declined from a pretest mean of 129.73 (SD = 6.16) to a posttest mean of 44.97 (SD = 9.08), a mean decrease of 84.76 points, which corresponds to a mean improvement in cognitive functioning through reduced negative automatic thoughts.

**Table 1:** Descriptive statistics of pretest and posttest scores of DERS and CSATQ by group (*N* = 128)*.*

Group	*N*	DERSpre mean (SD)	DERSpost mean (SD)	Mean Diff	CSATQpre mean (SD)	CSATQpost mean (SD)	Mean Diff
Treatment (CBT-journalling)	63	152.03 (7.12)	72.44 (9.25)	**–79.59**	129.73 (6.16)	44.97 (9.08)	**–84.76**
Control (no treatment)	65	151.62 (7.05)	135.78 (30.27)	**–15.83**	130.35 (6.09)	104.22 (40.40)	**–26.14**
Overall	128	151.82 (7.06)	104.61 (38.91)	**–47.21**	130.05 (6.11)	75.05 (41.80)	**–54.99**

CBT = cognitive behavioral therapy, CSATQ = clothing style automatic thoughts questionnaire, DERS = difficulties in emotion regulation scale.

In contrast, the no-treatment control group showed smaller changes. The DERS score declined from a pretest mean of 151.62 (SD = 7.05) to 135.78 (SD = 30.27) at posttest, a mean reduction of 15.83 points, indicating a minimal gain in emotional regulation. Likewise, the CSATQ scores dropped from 130.35 (SD = 6.09) at pretest to 104.22 (SD = 40.40) at posttest, with a mean reduction of 26.14 points, representing a modest cognitive improvement.

Across both groups combined, the overall DERS scores declined from 151.82 (SD = 7.06) at pretest to 104.61 (SD = 38.91) at posttest, indicating a mean reduction of 47.21 points. Similarly, the overall CSATQ scores decreased from a mean of 130.05 (SD = 6.11) to 75.05 (SD = 41.80), reflecting a reduction of 54.99 points. These overall reductions in DERS and CSATQ scores suggest that participation in CBT-based journaling was associated with greater mean gains in emotional and cognitive functioning, as evidenced by lower scores on both measures.

## 5. Hypothesis testing

### 5.1. Hypothesis testing: emotional responses (DERS)

Results from the analysis of covariance (ANCOVA) presented in Table 2 indicate a statistically significant main effect of therapy group on participants’ posttest scores on the difficulties in emotion regulation scale (DERS), *F*(1, 125) = 253.10, *P* < .001, partial η^2^ = .669. This suggests that after adjusting for baseline emotional regulation scores (DERSpre), the type of intervention (CBT-based journaling vs no treatment) accounted for approximately 66.9% of the variance in emotional response outcomes, a large effect size according to Cohen (1988) criteria.

**Table 2:** Analysis of covariance (ANCOVA) of the effect of CBT-based journaling on participants’ emotional responses to clothing styles measured by DERS.

Source	Type III sum of squares	DF	Mean square	*F*	Sig.	Partial eta squared
Corrected model	129,310.596*	2	64,655.298	128.264	.000	.672
Intercept	7297.907	1	7297.907	14.478	.000	.104
DERSpre	958.668	1	958.668	1.902	.170	.015
Therap	127,584.255	1	127,584.255	253.104	.000	.669
Error	63,009.872	125	504.079			
Total	1,593,040.000	128				
Corrected total	192,320.469	127				

CBT = cognitive behavioral therapy, DERS = difficulties in emotion regulation scale.

* R squared = .672 (adjusted R squared = .667).

The covariate, DERSpre, was not a statistically significant predictor of posttest DERS scores, *F*(1, 125) = 1.90, *P* = .170, partial η^2^ = .015, indicating that baseline emotional regulation did not significantly influence post-intervention outcomes. However, the overall ANCOVA model was statistically significant, *F*(2, 125) = 128.26, *P* < .001, and explained 67.2% of the variance in emotional responses as measured by the DERS (*R*^2^ = .672, Adjusted *R*^2^ = .667).

These findings provide strong evidence that CBT-based journaling was effective in improving emotional regulation related to participants’ responses to clothing styles. Accordingly, the null hypothesis, which stated that CBT-based journaling would have no significant effect on emotional responses to clothing styles, is rejected. The data support the alternative hypothesis and affirm the efficacy of the CBT-based journaling intervention in fostering healthier emotional outcomes.

### 5.2. Hypothesis testing: irrational beliefs (CSATQ)

As presented in Table 3, the analysis of covariance (ANCOVA) revealed a statistically significant main effect of the therapy group on participants’ posttest scores on the automatic thoughts questionnaire (CSATQ), *F*(1, 125) = 127.82, *P* < .001, partial η^2^ = .506. This indicates that the CBT-based journaling intervention accounted for approximately 50.6% of the variance in post-intervention irrational beliefs related to clothing styles, representing a large effect size.^[44]^

**Table 3:** Analysis of covariance (ANCOVA) of the effect of CBT-based journaling on participants’ irrational belief to clothing styles measured by CSATQ.

Source	Type III sum of squares	DF	Mean square	*F*	Sig.	Partial eta squared
Corrected model	112,301.136*	2	56,150.568	64.053	.000	.506
Intercept	1647.818	1	1647.818	1.880	.173	.015
CSATQpre	1.440	1	1.440	.002	.968	.000
Therap	112,045.998	1	112,045.998	127.816	.000	.506
Error	109,577.482	125	876.620			
Total	942,929.000	128				
Corrected total	221,878.617	127				

CBT = cognitive behavioral therapy, CSATQ = clothing style automatic thoughts questionnaire.

* R squared = .506 (adjusted R squared = .498).

The covariate, CSATQpre, was not a statistically significant predictor of posttest scores, *F*(1, 125) = 0.002, *P* = .968, partial η^2^ = .000, suggesting that participants’ baseline irrational beliefs had no meaningful influence on their post-intervention outcomes. Despite this, the overall ANCOVA model was statistically significant, *F*(2, 125) = 64.05, *P* < .001, explaining approximately 50.6% of the variance in CSATQ posttest scores (*R*^2^ = .506, Adjusted *R*^2^ = .498).

These findings provide substantial evidence that CBT-based journaling effectively reduced irrational beliefs related to clothing styles. As such, the null hypothesis, which posited no significant difference in post-intervention irrational beliefs between groups, is rejected. The results affirm the intervention’s value in improving participants’ cognitive processing of clothing-related thoughts, aligning with the study’s second objective.

## 6. Discussion

This study provides robust evidence supporting the effectiveness of CBT-based journaling in improving adolescents’ emotional responses to their clothing styles. Participants who practiced CBT-based journaling demonstrated notable enhancements in emotional response and a decrease in irrational beliefs related to clothing styles. These results are consistent with the theoretical principles of CBT, which suggest that systematic cognitive and reflective exercises can aid in adaptive emotional processing, challenge maladaptive thoughts, and promote a positive self-image.^[45,46]^ A key finding of this research was the marked enhancement in emotional response within the intervention group. This aligns with earlier studies indicating that CBT interventions, including journaling, foster emotional awareness and effective regulation techniques by prompting individuals to recognize, express, and reframe maladaptive emotional responses.^[46–48]^ The significant reduction in scores on the difficulties in emotion regulation scale (DERS) implies that CBT-based journaling could be an effective, scalable method for assisting adolescents in navigating the emotional difficulties linked to self-presentation and social assessment.^[46,49]^ Additionally, the notable decline in irrational beliefs concerning clothing selections, as assessed by the automatic thoughts questionnaire, highlights the cognitive advantages of CBT-based journaling, which provides a structured approach for adolescents to confront and alter negative self-dialogue and distorted perceptions.^[50–52]^ The intervention’s influence on cognitive restructuring is further corroborated by meta-analytic findings that demonstrate CBT’s consistent ability to diminish irrational beliefs and related emotional distress.^[53]^ This study contributes to the existing body of literature by illustrating that CBT-based journaling serves as a potent and accessible intervention for enhancing emotional response and diminishing irrational beliefs.^[54–56]^ The results advocate for the incorporation of journaling into school-based and clinical programs addressing appearance-related issues and propose directions for future research, including the investigation of long-term effects and the modification of journaling protocols for various cultural and developmental settings.^[57,58]^

The findings of this study have important theoretical and practical implications. The observed improvement in emotional responses and reduction in irrational beliefs among adolescents who engaged in CBT-based journaling supports the central premise of cognitive-behavioral theory that systematic cognitive reflection can enhance emotional regulation and cognitive restructuring. This suggests that CBT-based journaling is not only effective but also flexible and low-cost, making it an ideal intervention for addressing appearance-related concerns in school or community settings. It empowers adolescents to independently manage distressing thoughts associated with clothing and self-image, promoting self-awareness and healthier emotional processing during a critical stage of identity formation.

Furthermore, the results extend existing knowledge by highlighting the cognitive and emotional benefits of adapting CBT techniques to specific social contexts, such as clothing and appearance. The reduction in irrational clothing-related beliefs, as captured by the CSATQ, emphasizes the intervention’s relevance for addressing fashion-induced cognitive distortions. This opens up new avenues for mental health education and preventive strategies that leverage journaling as a structured yet personal tool for psychological resilience, particularly among adolescents vulnerable to social judgment and appearance anxiety.

Despite its strengths, this study has several limitations. First, the reliance on self-report measures may introduce response bias, as participants might have over- or under-reported their emotional experiences and thought patterns. Second, the relatively short duration of the intervention limits conclusions about the long-term sustainability of the observed improvements. Third, while the adapted CSATQ demonstrated strong face validity, further psychometric validation, including factor analysis and test-retest reliability, would strengthen its utility for broader research and clinical use.

Additionally, the sample was limited to adolescents within a specific geographical region, which may affect the generalizability of the findings. Future studies should involve more diverse samples across different regions and socio-cultural backgrounds to examine whether the effectiveness of CBT-based journaling varies by context. Including follow-up assessments would also help in determining whether changes in emotional response and cognitive distortions are maintained over time.

Based on the positive outcomes observed, it is recommended that CBT-based journaling be integrated into school counseling programs, mental health curricula, and youth empowerment initiatives. Educational institutions can adopt structured journaling sessions as part of life skills or psychosocial support programs, enabling students to build self-reflective habits and emotional awareness. Mental health practitioners working with adolescents should consider incorporating journaling assignments into therapy or psychoeducational groups to support the development of adaptive self-dialogue and self-expression.

In addition, further research should explore the long-term effects of CBT-based journaling on emotional regulation and cognitive distortions. Investigating whether sustained journaling practice leads to lasting changes in self-image and emotional well-being would provide valuable insights. There is also a need to culturally adapt and test journaling protocols across diverse populations to ensure relevance and inclusivity. Exploring digital journaling tools and their effectiveness may also offer scalable options for wider implementation, particularly in underserved or resource-constrained settings.

## 7. Conclusion

This research illustrates that CBT-based journaling serves as an effective and accessible method for enhancing the emotional responses of adolescents towards their clothing styles. The intervention resulted in notable improvements in emotional response and a decrease in irrational beliefs, with these advantages evident irrespective of clothing styles. These results underscore the importance of self-directed cognitive-behavioral strategies in promoting the well-being of adolescents and indicate that journaling can be easily incorporated into educational and community initiatives. By enabling adolescents to process their emotions and confront negative self-perceptions, CBT-based journaling provides a pragmatic means of cultivating resilience and a positive self-image during a crucial stage of development.

## Author contributions

**Conceptualization:** Ngozi Ngwanma Awoke, Daniel Okon.

**Data curation:** Uzoamaka C. Ogor, Damian C. Ncheke, Ngozi Ngwanma Awoke, Nkechi T. Egenti, Chinonye Lilian Opara, Daniel Okon.

**Formal analysis:** Damian C. Ncheke, Lilian C. Ozoemena, Nkechi T. Egenti, Patience Nwobodo, Daniel Okon.

**Funding acquisition:** Uzoamaka C. Ogor, Nkechi T. Egenti, Daniel Okon.

**Investigation:** Uzoamaka C. Ogor, Damian C. Ncheke, Lilian C. Ozoemena, Dorathy C. Ezeunara, Patience Nwobodo.

**Methodology:** Ekwutosi Beatrice Oluah, Uzoamaka C. Ogor, Uche Hope Ekwueme, Nkechi T. Egenti, Chinonye Lilian Opara, Patience Nwobodo, Blessed Samuel Okechukwu Efido, Daniel Okon.

**Project administration:** Ekwutosi Beatrice Oluah, Uche Hope Ekwueme, Chinonye Lilian Opara.

**Resources:** Ekwutosi Beatrice Oluah, Uzoamaka C. Ogor, Ngozi Ngwanma Awoke, Uche Hope Ekwueme, Nkechi T. Egenti, Dorathy C. Ezeunara, Patience Nwobodo.

**Software:** Damian C. Ncheke, Uche Hope Ekwueme, Chinonye Lilian Opara.

**Supervision:** Ekwutosi Beatrice Oluah, Lilian C. Ozoemena, Blessed Samuel Okechukwu Efido.

**Validation:** Ekwutosi Beatrice Oluah, Damian C. Ncheke, Ngozi Ngwanma Awoke, Dorathy C. Ezeunara, Patience Nwobodo, Blessed Samuel Okechukwu Efido.

**Visualization:** Ekwutosi Beatrice Oluah, Lilian C. Ozoemena, Ngozi Ngwanma Awoke, Chinonye Lilian Opara, Blessed Samuel Okechukwu Efido, Daniel Okon.

**Writing – original draft:** Uche Hope Ekwueme, Daniel Okon.

**Writing – review & editing:** Lilian C. Ozoemena, Dorathy C. Ezeunara, Patience Nwobodo, Blessed Samuel Okechukwu Efido.

## References

[1] Nobre J Calha A Luis H. Mental health literacy and positive mental health in adolescents: a correlational study. Int J Environ Res Public Health. 2022;19:8165. 35805824 10.3390/ijerph19138165 PMC9266633

[2] da Nunes C. The Importance on self-expression through clothing and fashion: a view on digital identity and digital fashion [Master’s dissertation, University of Beira Interior]. uBibliorum; 2023. http://hdl.handle.net/10400.6/13583.

[3] Moody W Kinderman P Sinha P. An exploratory study. J Fashion Marketing Manag. 2010;14:161–79.

[4] Li W Huang Z Liu C. Teaching exploration of clothing fabric irrational design application project based on working process and emotional behavior change. Int J Neuropsychopharmacol. 2022;25(Supplement 1): A79–80.

[5] Meinert MV. Garments as healing spaces: Conceptual clothing for emotional regulation, connection and resilience [Master of Fine Arts thesis, The University of Texas at Austin]. Texas ScholarWorks; 2023. https://repositories.lib.utexas.edu/items/e5ff0f83-0d04-426e-b15a-abc32cde4a46.

[6] Lindeman R Desmet P Filippi M. Wearing black when feeling blue: an exploration of the relationship between clothing and mood. IASDR 2023. 2023. https://dl.designresearchsociety.org/iasdr/iasdr2023/fullpapers/186/. Toegang verkry Mei 19, 2025

[7] Sebri V Pravettoni G. Tailored psychological interventions to manage body image: an opinion study on breast cancer survivors. Int J Environ Res Public Health. 2023;20:2991. 36833684 10.3390/ijerph20042991 PMC9957299

[8] Veron-Guidry S Williamson DA Netemeyer RG. Structural modeling analysis of body dysphoria and eating disorder symptoms in preadolescent girls. Eat Disord. 1997;5:15–27.

[9] Bernstein EE Bentley KH Nock MK Stein MB Beck S Kleiman EM. An ecological momentary intervention study of emotional responses to smartphone-prompted CBT skills practice and the relationship to clinical outcomes. Behav Ther. 2022;53:267–80. 35227403 10.1016/j.beth.2021.09.001 PMC8891654

[10] Jazaieri H Morrison AS Goldin PR Gross JJ. The role of emotion and emotion regulation in social anxiety disorder. Curr Psychiatry Rep. 2015;17:531. 25413637 10.1007/s11920-014-0531-3

[11] Aupperle RL Allard CB Simmons AN. Neural responses during emotional processing before and after cognitive trauma therapy for battered women. Psychiatry Res. 2013;214:48–55. 23916537 10.1016/j.pscychresns.2013.05.001

[12] Maeng L Chae J Oh K. The influence of clothing color preference of adolescents on the self expression desire and fashion interest. Korean J Human Ecol. 2009;18:1077–86.

[13] Maxey G. Fashion psychology: the relationship between clothing and self. Counseling Family Ther Scholarship Rev. 2022;4:1.

[14] Stolovy T. Befriending the body through clothes: the role of clothing in secular and religious women’s body appreciation. Front Psychol. 2024;15:1297663. 38873515 10.3389/fpsyg.2024.1297663 PMC11172143

[15] Hayde S. Am I what I wear? The relationship between clothing, mood enhancement and self-concept clarity [Undergraduate dissertation, Institute of Art, Design and Technology]. Illustro Research Repository; 2021. https://hdl.handle.net/10779/iadt.25189088

[16] Scott J. Fashion and positive psychology: Interactions between clothing, mood, self-concept, and well-being [Master of Arts major research paper, Ryerson University]. RShare, Toronto Metropolitan University; 2023. 10.32920/ryerson.14649540

[17] Williams A Grisham JR. Impulsivity, emotion regulation, and mindful attentional focus in compulsive buying. Cognit Ther Res. 2012;36:451–7.

[18] Tylka TL Wood-Barcalow NL. The body appreciation scale-2: item refinement and psychometric evaluation. Body Image. 2014;12:53–67. 25462882 10.1016/j.bodyim.2014.09.006

[19] Twenge JM. iGen: Why today’s super-connected kids are growing up less rebellious, more tolerant, less happy – and completely unprepared for adulthood. Atria Books; 2019.

[20] Ziółkowski A Rynkiewicz A. Generational challenges in emotional development in the digital era: implications for Generation Z and Alpha. Front Sport Act Living. 2024;6:1416154.

[21] Nowak M Wysocki P. Threats to the psychosocial development of Generation Alpha in the digital age. Arch Budo. 2024;20:1–12.

[22] Vogel EA Rose JP Roberts LR Eckles K. Social comparison, social media, and self-esteem. Psychol Popular Media Culture. 2014;3:206–22.

[23] Yahaya Nasidi Q Hassan I Ahmad MF. Mediating role of social media in the relationship between reliability, perceived usefulness on online shopping behaviour: building a conceptual framework. Int J Acad Res Bus Soc Sci. 2021;11:385.

[24] de Klerk H Tselepis T. The early‐adolescent female clothing consumer: expectations, evaluation and satisfaction with fit as part of the appreciation of clothing quality. J Fashion Marketing Manag. 2007;11:413–28.

[25] Song K. The effects of perceptual body image and appearance management behavior on clothing behaviors. J Korean Soc Clothing Textiles. 2009;33:1611–21.

[26] De Castella K Goldin P Jazaieri H Heimberg RG Dweck CS Gross JJ. Emotion beliefs and cognitive behavioural therapy for social anxiety disorder. Cogn Behav Ther. 2015;44:128–41. 25380179 10.1080/16506073.2014.974665

[27] Mirchandaney R Barete R Asarnow LD. Moderators of cognitive behavioral treatment for insomnia on depression and anxiety outcomes. Curr Psychiatry Rep. 2022;24:121–8. 35061137 10.1007/s11920-022-01326-3 PMC8948126

[28] Altmann U Schoenherr D Paulick J. Associations between movement synchrony and outcome in patients with social anxiety disorder: evidence for treatment specific effects. Psychother Res. 2020;30:574–90. 31213149 10.1080/10503307.2019.1630779

[29] Spain D Sin J Harwood L Mendez MA Happé F. Cognitive behaviour therapy for social anxiety in autism spectrum disorder: a systematic review. AIA. 2017;3:34–46.

[30] Ghadakzadeh S Ghazipour A Khajeddin N Karimian N Borhani M. Body Image Concern Inventory (BICI) for identifying patients with BDD seeking rhinoplasty: using a Persian (Farsi) version. Aesthetic Plast Surg. 2011;35:989–94. 21491168 10.1007/s00266-011-9718-8

[31] Clarke A Thompson AR Jenkinson E Rumsey N Newell R. CBT for appearance anxiety: Psychosocial interventions for anxiety due to visible difference. John Wiley & Sons; 2013.

[32] Alessi J de Oliveira GB Franco DW. Mental health in the era of COVID-19: prevalence of psychiatric disorders in a cohort of patients with type 1 and type 2 diabetes during the social distancing. Diabetol Metab Syndr. 2020;12:76. 32879637 10.1186/s13098-020-00584-6 PMC7457442

[33] Hirshfeld-Becker DR Masek B Henin A. Cognitive behavioral therapy for 4- to 7-year-old children with anxiety disorders: a randomized clinical trial. J Consult Clin Psychol. 2010;78:498–510. 20658807 10.1037/a0019055

[34] Aguilar-Yamuza B Trenados Y Herruzo C Pino MJ Herruzo J. A systematic review of treatment for impulsivity and compulsivity. Front Psychiatry. 2024;15:1430409. 39391084 10.3389/fpsyt.2024.1430409 PMC11465090

[35] Ghosh P Bhattacharyya R. Mood journaling: a new Year\’s journey to track emotions. Indian J Private Psychiatry. 2025;19:1–2.

[36] Chatwin H Stapleton P Porter B Devine S Sheldon T. The effectiveness of cognitive behavioral therapy and emotional freedom techniques in reducing depression and anxiety among adults: a pilot study. Integr Med (Encinitas). 2016;15:27–34. PMC4898279 27330487

[37] Upadhyay N Kamble A. The role of environmental concerns and self-expression in ethical fashion consumption: a mediated model of consumer values. J Global Marketing. 2025;38:487–509.

[38] Menon S Bhagat V. Literature study on the efficacy of antidepressants with CBT in the treatment of depression. RJPT. 2022;15:2775–87.

[39] Villabø MA Narayanan M Compton SN Kendall PC Neumer SP. Cognitive-behavioral therapy for youth anxiety: an effectiveness evaluation in community practice. J Consult Clin Psychol. 2018;86:751–64. 30138014 10.1037/ccp0000326 PMC6108446

[40] Faul F Erdfelder E Buchner A Lang AG. Statistical power analyses using G*Power 3.1: tests for correlation and regression analyses. Behav Res Methods. 2009;41:1149–60. 19897823 10.3758/BRM.41.4.1149

[41] Gratz KL Roemer L. Multidimensional assessment of emotion regulation and dysregulation: development, factor structure, and initial validation of the difficulties in emotion regulation scale. J Psychopathol Behav Assess. 2004;26:41–54.

[42] Hollon S Kendall PC. Cognitive self-statements in depression: development of an automatic thoughts questionnaire. Cognitive Ther Res. 1980;4:383–95.

[43] Cully JA Teten AL. A Therapists Guide to Brief CBT. Department of Veterans Affairs, South Central Mental Illness Research, Education, and Clinical Center (MIRECC); 2008. papers3://publication/uuid/511A96F5-439A-403D-BB8C-C85BE1A99789

[44] Cohen J. Statistical power analysis for the behavioral sciences. 2nd ed.). Lawrence Erlbaum Associates.; 1988.

[45] Baker R Owens M Thomas S. Does CBT facilitate emotional processing? Behav Cogn Psychother. 2012;40:19–37. 21392415 10.1017/S1352465810000895

[46] Aldao A Jazaieri H Goldin PR Gross JJ. Adaptive and maladaptive emotion regulation strategies: interactive effects during CBT for social anxiety disorder. J Anxiety Disord. 2014;28:382–9. 24742755 10.1016/j.janxdis.2014.03.005 PMC4089517

[47] Suveg C Sood E Comer JS Kendall PC. Changes in emotion regulation following cognitive-behavioral therapy for anxious youth. J Clin Child Adolesc Psychol. 2009;38:390–401. 19437299 10.1080/15374410902851721

[48] Pacheco DC de Serpa Arruda Moniz AID Caldeira SN Silva ODL. Online impulse buying—integrative review on self-regulation, risks and self-regulatory strategies. Springer. 2022;284:311–9.

[49] Rubin-Falcone H Weber J Kishon R. Neural predictors and effects of cognitive behavioral therapy for depression: the role of emotional reactivity and regulation. Psychol Med. 2020;50:146–60. 30739618 10.1017/S0033291718004154

[50] Turner MJM Martin J. Rational emotive behavior therapy (REBT), irrational and rational beliefs, and the mental health of athletes. Front Psychol. 2016;7:1423. 27703441 10.3389/fpsyg.2016.01423 PMC5028385

[51] Turner M Barker JB. Examining the efficacy of rational-emotive behavior therapy (REBT) on irrational beliefs and anxiety in elite youth cricketers. J Appl Sport Psychol. 2013;25:131–47.

[52] Kaplan V Düken ME Kaya R Almazan J. Investigating the effects of cognitive-behavioral-therapy-based psychoeducation program on university students’ automatic thoughts, perceived stress, and self-efficacy levels. J Res Heal. 2023;13:87–98.

[53] Vîslă A Flückiger C Grosse Holtforth M David D. Irrational beliefs and psychological distress: a meta-analysis. Psychother Psychosom. 2016;85:8–15. 26609889 10.1159/000441231

[54] Outar L. Body image beliefs: The examination of rational emotive behaviour therapy (rebt) and assessment of irrational and rational beliefs in body image. Staffordshire University; 2022. http://eprints.staffs.ac.uk/7668/ Toegang verkry Mei 18, 2025

[55] Miller AJ Turner MJ Ollier W Hattersley AA. Understanding the influence of irrational beliefs and body image inflexibility on exercise dependence and psychological well-being: a latent profile analysis approach. J Sports Sci. 2023;41:291–7. 37163462 10.1080/02640414.2023.2208952

[56] Cengiz H Barin A. How does body appreciation affect maladaptive consumption through fashion clothing involvement? A multi-group analysis of gender. J Fashion Marketing Manag. 2024;29:94.

[57] King AM Plateau CR Turner MJ Young P Barker JB. A systematic review of the nature and efficacy of rational emotive behaviour therapy interventions. PLoS One. 2024;19:e0306835. 38980891 10.1371/journal.pone.0306835 PMC11232995

[58] Outar L Turner MJ Wood AG O’Connor H. Muscularity rationality: an examination of the use of Rational Emotive Behaviour Therapy (REBT) upon exercisers at risk of muscle dysmorphia. Psychol Sport Exercise. 2021;52:101813.

